# Supplementary material for: Yiai Fuzheng decoction inhibits triple-negative breast cancer by remodeling the immune microenvironment
Source: Front Immunol. 2025 Sep 30;16:1615631. doi: 10.3389/fimmu.2025.1615631 (PMC12518410; doi:10.3389/fimmu.2025.1615631)
Supplement: Supplementary file 2 [file DataSheet1.zip › Supplementary File 2/Treatment/C_vs_D_vs_H/06_Pathway_Analysis/Pathway_Analysis/Sig_Pathways_Name/Caffeine metabolism.pdf]

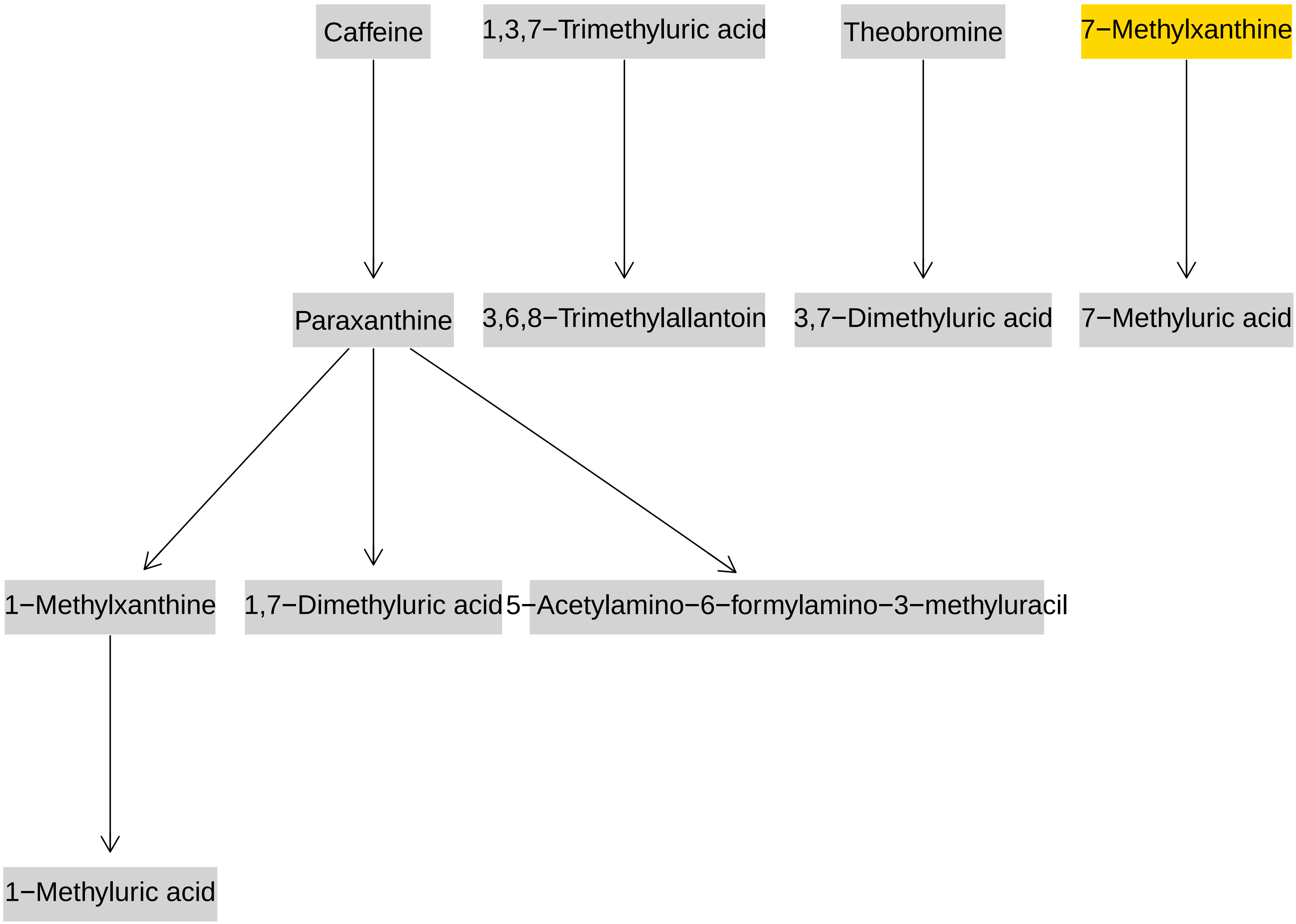
Caffeine
1,3,7−Trimethyluric acid
Theobromine
7−Methylxanthine
Paraxanthine
3,6,8−Trimethylallantoin
3,7−Dimethyluric acid
7−Methyluric acid
1−Methylxanthine
1,7−Dimethyluric acid
5−Acetylamino−6−formylamino−3−methyluracil
1−Methyluric acid
